# Xeroderma Pigmentosum Group A Suppresses Mutagenesis Caused by Clustered Oxidative DNA Adducts in the Human Genome

**DOI:** 10.1371/journal.pone.0142218

**Published:** 2015-11-11

**Authors:** Akira Sassa, Nagisa Kamoshita, Yuki Kanemaru, Masamitsu Honma, Manabu Yasui

**Affiliations:** Division of Genetics and Mutagenesis, National Institute of Health Sciences, Setagaya-ku, Tokyo, Japan; University of South Alabama Mitchell Cancer Institute, UNITED STATES

## Abstract

Clustered DNA damage is defined as multiple sites of DNA damage within one or two helical turns of the duplex DNA. This complex damage is often formed by exposure of the genome to ionizing radiation and is difficult to repair. The mutagenic potential and repair mechanisms of clustered DNA damage in human cells remain to be elucidated. In this study, we investigated the involvement of nucleotide excision repair (NER) in clustered oxidative DNA adducts. To identify the *in vivo* protective roles of NER, we established a human cell line lacking the NER gene xeroderma pigmentosum group A (*XPA*). *XPA* knockout (KO) cells were generated from TSCER122 cells derived from the human lymphoblastoid TK6 cell line. To analyze the mutagenic events in DNA adducts *in vivo*, we previously employed a system of tracing DNA adducts in the targeted mutagenesis (TATAM), in which DNA adducts were site-specifically introduced into intron 4 of thymidine kinase genes. Using the TATAM system, one or two tandem 7,8-dihydro-8-oxoguanine (8-oxoG) adducts were introduced into the genomes of TSCER122 or *XPA* KO cells. In *XPA* KO cells, the proportion of mutants induced by a single 8-oxoG (7.6%) was comparable with that in TSCER122 cells (8.1%). In contrast, the lack of XPA significantly enhanced the mutant proportion of tandem 8-oxoG in the transcribed strand (12%) compared with that in TSCER122 cells (7.4%) but not in the non-transcribed strand (12% and 11% in *XPA* KO and TSCER122 cells, respectively). By sequencing the tandem 8-oxoG-integrated loci in the transcribed strand, we found that the proportion of tandem mutations was markedly increased in *XPA* KO cells. These results indicate that NER is involved in repairing clustered DNA adducts in the transcribed strand *in vivo*.

## Introduction

Genomic DNA is constantly exposed to both exogenous and endogenous genotoxic agents. Among them, ionizing radiation (IR) induces various DNA adducts in the genome because of its ability to produce reactive oxygen species in cells. Moreover, IR induces clustered DNA damage, which is defined as multiple DNA damage sites [oxidized DNA adducts, apurinic/apyrimidinic (AP) sites, or strand breaks] within one or two helical turns of the duplex DNA. Non-double-strand break (DSB) clustered DNA damage comprises 70%–80% of the complex DNA damage induced by IR, whereas DSB accounts for 20%–30% [1, 2]. Clustered DNA damage is considered to be more difficult to repair than a single DNA damage site. Unrepaired damage contributes to mutagenesis, cancer development, and disease [3].

7,8-Dihydro-8-oxoguanine (8-oxoG) is one of the major oxidative DNA adducts induced by IR. Because of the altered hydrogen-bonding potential, 8-oxoG can pair with an adenine during replication [4] and cause G·C to T·A transversion mutations. 8-OxoG is primarily repaired by the base excision repair (BER) pathway [5]. In mammalian cells, 8-oxoG paired with cytosine is readily repaired by 8-oxoguanine DNA glycosylase (OGG1)-initiated BER [6]. Furthermore, repair can occur via another BER pathway in which the human *MutY* homolog (MYH) removes an adenine from the 8-oxoGA mispair [7]. However, it is more challenging to repair 8-oxoG in clustered DNA damage sites via BER.

Numerous studies have investigated BER retardation at clustered DNA damage sites that results in enhanced genomic instability. Different types of damage (a thymine glycol, an AP site, a single-strand break, or a mismatched base-pair) adjacent to 8-oxoG strongly inhibits 8-oxoG excision by OGG1 [8–10]. When two 8-oxoG are located in tandem nucleotides on the same strand, the repair of these adducts is also delayed [11]. DNA damage in close opposition to an 8-oxoG also inhibits 8-oxoG repair [12–14]. The biological relevance of these clustered damages in DNA has been extensively investigated in both *Escherichia coli* and yeast [15–24]. However, although several studies have examined the mutagenic events of clustered oxidative damage to episomal DNA in mammalian cells [25, 26], these repair mechanisms in the human genome are still not well understood.

A few previous reports have indicated that nucleotide excision repair (NER), which repairs bulky DNA adducts (such as cyclobutane pyrimidine dimers), is involved in the removal of oxidative DNA adducts. An *in vitro* study demonstrated that NER recognizes 8-oxoG in oligonucleotides [27]. A high-sensitivity method that combined single-cell gel electrophoresis with fluorescence *in situ* hybridization also revealed 8-oxoG removal from the transcribed strand (TS) of DNA by transcription-coupled NER [28]. On the basis of these studies, we posed the following question: what role does NER play in the suppression of mutagenesis induced by a single and/or clustered 8-oxoG formed in the genome?

Here we established a human cell line lacking xeroderma pigmentosum complementation group A (*XPA*), a gene essential for NER on both TS and the non-transcribed strand (NTS) [29]. We previously developed a unique system for tracing DNA adducts in targeting mutagenesis (TATAM) by introducing a DNA adduct site specifically into intron 4 of the thymidine kinase (*TK*) gene in human lymphoblastoid cells [30]. Using the TATAM system, either one or two tandemly located 8-oxoG were introduced into the genome of the wild-type or *XPA* knockout (KO) cells for analysis of the mutagenic potential of adducts. Our findings indicate that NER is a possible repair mechanism of clustered oxidative DNA adducts particularly in TS of the human genome.

## Materials and Methods

### Cell culture

Human lymphoblastoid TSCER122 cells, which were derived from TK6 cells [31], have been previously described [30]. Cells were cultured in RPMI 1640 (Nacalai Tesque) with 10% heat-inactivated horse serum (JRH Biosciences), 200 μg/ml sodium pyruvate, 100 U/ml penicillin, and 100 μg/ml streptomycin at 37°C in an atmosphere of 5% CO_2_ and 100% humidity.

### Construction of XPA knockout cells

We purchased custom zinc finger nuclease (ZFN) for targeting *XPA* from Sigma-Aldrich. The design, assembly, and validation of ZFN were performed using the CompoZr® Custom ZFN Service (Sigma-Aldrich). The target sequence for ZFN, that is located in exon 1 of the *XPA* gene, was as follows: 5′-CAGGCCCGGCTGGCTGCCCggcccTACTCGGCGACGGCGGCT-3′. ZFN mRNA (2 μg) was transfected into TSCER122 (5 × 10^6^) cells supplemented with 0.1 ml Nucleofector solution V (Lonza) using Nucleofector I according to the manufacturer’s recommendations. After 24 h in culturing medium, cells were seeded into 96-microwell plates at 1.6 cells/well (i.e., 8 cells/ml) and then incubated at 37°C for 7–10 days. Genomic DNA was isolated from colonies and subjected to PCR using KOD FX (Toyobo) with the primers 5′-AGCTAGGTCCTCGGAGTGG-3′ and 5′-GGACAGGACGCTTTGACAAG-3′. The amplified DNA fragment was then sequenced to confirm deletion around the ZFN target site.

### Western blot analysis

Total cell extracts were prepared from exponentially growing cells and were subjected to electrophoresis in 10% SDS-polyacrylamide gel and then transferred onto a PVDF membrane. The membrane was blocked with 5% skim milk. To detect XPA, APE1, or α-tubulin, the membrane was incubated in 1:100 dilution of anti-XPA monoclonal antibody (ab2352, Abcam), 1:2000 dilution of anti-APE1 monoclonal antibody (ab194, Abcam), or 1:10000 dilution of anti-α-tubulin monoclonal antibody (ab7291, Abcam) overnight. After washing with phosphate-buffered saline containing 0.05% Tween 20, the membrane was incubated with 1:2500 dilution of anti-mouse IgG conjugated to horseradish peroxidase (GE Healthcare Bio-Sciences). To detect OGG1 or DNA polymerase β (Pol β), the membrane was incubated with 1:10000 dilution of anti-OGG1 monoclonal antibody (ab124741, Abcam) or 1:1000 dilution of anti-Pol β polyclonal antibody (ab26343, Abcam) overnight. After washing with phosphate-buffered saline containing 0.05% Tween 20, the membrane was incubated with 1:2500 dilution of anti-rabbit IgG conjugated to horseradish peroxidase (GE Healthcare Bio-Sciences). The proteins were visualized by chemiluminescence using the ECL system (GE Healthcare Bio-Sciences).

### Irradiation conditions

Prior to irradiation with 254 nm ultraviolet (UVC), cells were washed and resuspended with RPMI 1640 without phenol red. Cells (2.5 × 10^6^) in 5 ml medium were exposed to UVC in a 35 × 100 mm petri dish using a UV germicidal lamp at a fluence of 2.7 W/m^2^, which was monitored by a UV-light meter (Lutron). After irradiation, cells were seeded into 96-microwell plates at 1.6 cells/well (i.e., 8 cells/ml) to determine cell survival.

### PCR-based preparation of a site-specific modified targeting vector containing 8-oxoG adducts

**Fig 1:**
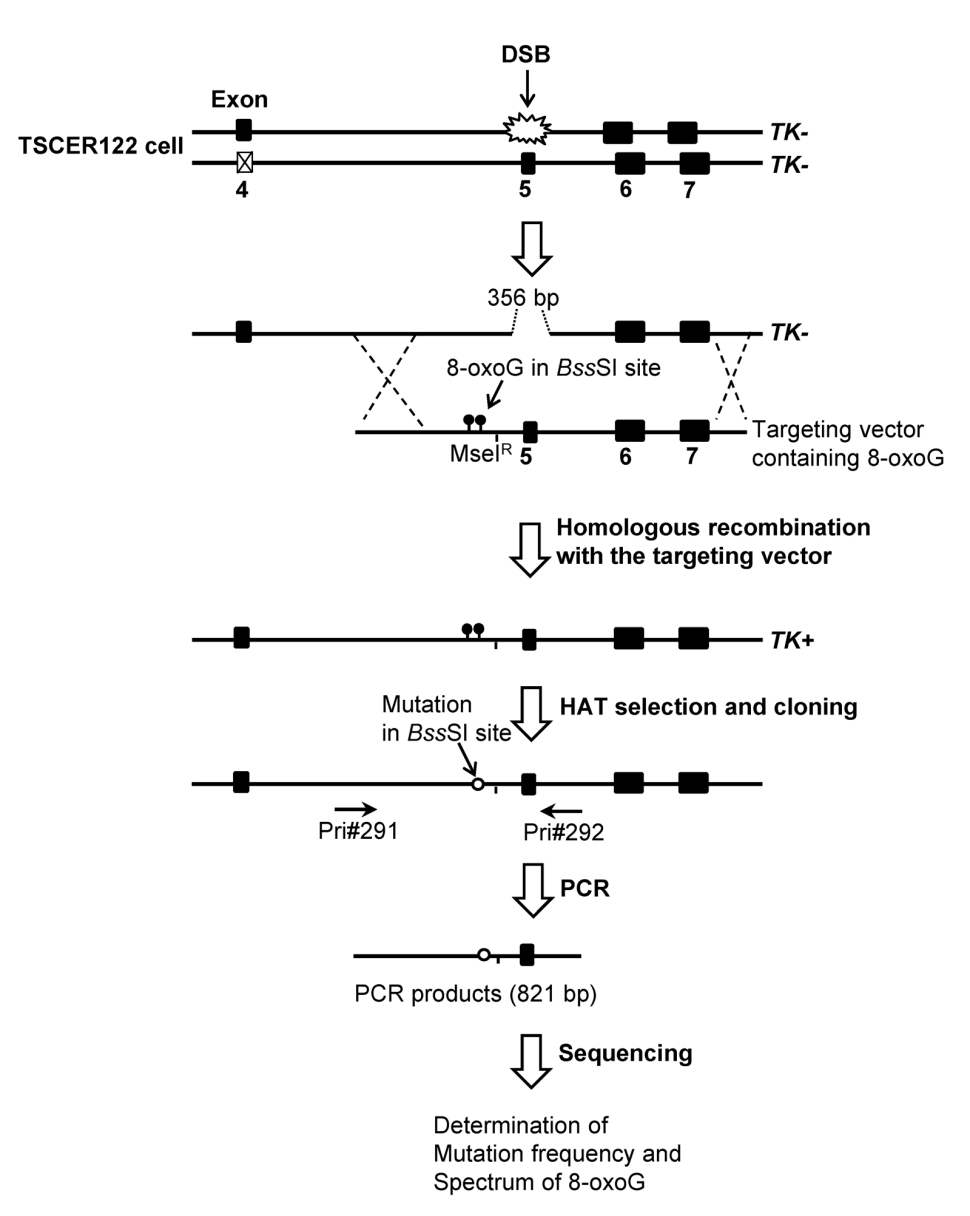
Overview of the TATAM system. Principle of the TATAM system. The targeting vector pvIT^G^, pvIT^1x8oG^, pvIT^2x8oG^, or pvINT^2x8oG^ and the I-SceI expression plasmid pCBASce were co-transfected into TSCER122 cells. DSB at the I-SceI site enabled high gene-targeting efficiency for the TATAM system by inducing DSB repair-enhanced site-specific homologous recombination. The targeting vector contained a *Mse*I^R^ site that was resistant to *Mse*I digestion and thereby distinguished between targeted and non-targeted revertants of *TK*. Genomic DNAs of the revertants were prepared, and part of the *TK* gene containing the DNA adduct-integrated site was amplified by PCR. The amplified fragment was sequenced as described in Materials and Methods.

Targeting vectors pvIT^1x8oG^, pvIT^2x8oG^, and pvINT^2x8oG^, carrying a single 8-oxoG in TS, two 8-oxoG in TS, and two 8-oxoG in NTS, respectively; the control vector pvIT^G^ without DNA adduct were prepared by a PCR-based method with the plasmid pTK15 as previously described [30, 32]. The targeting vector comprised 6.1 kb of the four individual *TK* genes, encompassing exons 5–7, and part of the I-SceI sequence in intron 4, which carries a loss-of-function TTAT deletion. We inserted an 8-oxoG into pvIT^1x8oG^ in place of the underlined guanine at the *Bss*SI site in TS (5′-CACGAG), two 8-oxoG into pvIT^2x8oG^ in TS (5′-CACGAG), and two 8-oxoG into pvINT^2x8oG^ in NTS (5′-CTCGTG) (S1A–S1C Fig). We labeled a 5′-TTCA-sequence (*Mse*I^R^) near the 8-oxoG-modified *Bss*SI site that was resistant to *Mse*I digestion and thereby distinguished between targeted and non-targeted revertants of *TK* according to interallelic recombination (Fig 1). The vectors were sequenced to confirm 8-oxoG presence at the expected site.

### Transfection and cloning of TK revertant cells

Prior to transfection, the concentrations of targeting vectors were determined with a Qubit® 2.0 Fluorometer with Qubit® dsDNA HS Assay Kit (Life technologies). The targeting vector (1 μg) and I-SceI expression plasmid pCBASce (50 μg) were co-transfected into 5 × 10^6^ cells that were suspended in 0.1 ml Nucleofector Solution V using Nucleofector I according to the manufacturer’s recommendations [33]. After 72 h of incubation, cells were seeded into 96-microwell plates in the presence of 200 μM hypoxanthine, 0.1 μM aminopterin, and 17.5 μM thymidine (HAT) in order to isolate 8-oxoG-integrated revertant clones. After 2 weeks of incubation, drug-resistant colonies (TK revertants) were counted.

### Mutation analysis

Genomic DNA templates for PCR were prepared from TK-revertant colonies using the alkaline lysis method according to a protocol provided by Toyobo Co., Ltd. Cells were incubated with 18 μl of 50 mM NaOH at 95°C for 10 min and then neutralized with the addition of 2 μl of 1 M Tris-HCl (pH 8.0). The resulting cell lysates were used as templates for PCR to amplify the *TK* gene fragments containing the 8-oxoG-integration site. PCR amplification was performed using KOD FX with forward and reverse primers: (Pri#291, intron 4), 5′-GCT CTT ACG GAA AAG GAA ACA GG-3′; (Pri#292, intron 5), 5′-CTG ATT CAC AAG CAC TGA AG-3′, respectively, as previously described [30]. Genomic regions around the *Bss*SI and *Mse*I^R^ sites were sequenced using an ABI 3730xl DNA analyzer (Applied Biosystems) and clones harboring the *Mse*I^R^ sequence were counted to determine the frequency of 8-oxoG integration and numbers of mutations at 12-bp (5′-TCC CAC GAG GCT) around the *Bss*SI site. The integration frequency of 8-oxoG adducts in the targeting vector was calculated by dividing the number of *Mse*I^R^ clones by the total number of revertant clones analyzed. Single-point mutation was defined as a single-base substitution, one-base insertion, or one-base deletion detected at an 8-oxoG. Tandem mutations were multiple-base substitutions, deletions, and/or insertions that were detected at sites, including 8-oxoG. Base substitutions, deletions, and/or insertions found at sites other than 8-oxoG were defined as non-targeted. Mutant proportions were calculated by dividing the number of mutants by the number of *Mse*I^R^-bearing clones.

### Complementation assay

For the complementation assay, the coding region of *XPA* was amplified from cDNA of TSCER122 cells by PCR with primers 5′-AGCTAGGTCCTCGGAGTGG-3′ and 5′-TTTTGAATTTTGAAAAGGACCAA-3′. The resulting fragment was further amplified by PCR with primers 5′-TGGGAATTCGCCACCATGGCGGCGGCCGA-3′ and 5′-TTTCAGTCGACTCACATTTTTTCATATGTCAGTT-3′. The PCR product was digested with *Eco*RI and *Sal*I and was cloned into the *Eco*RI/*Sal*I sites of pCl-neo vector (Promega). The obtained plasmid was named pCl-XPA, which contained *XPA* cDNA. *XPA* KO (5 × 10^6^) cells were transfected with 10 μg of *Bam*HI-linearlized pCl-XPA using Nucleofector I. After 48 h in the culturing medium, cells were seeded into 96-microwell plates in the presence of 1 mg/ml G418 (Roche). G418-resistant clones were then isolated and the expression of XPA was confirmed by Western blot analysis. The XPA-expressing clone was subjected to UV-irradiation and TATAM analyses.

### Statistical analysis

Statistical significance was evaluated by Student’s *t*-test or two-tailed Fisher’s exact test; P < 0.05 was considered to be significant.

## Results

### Establishment of XPA knockout cells

**Fig 2:**
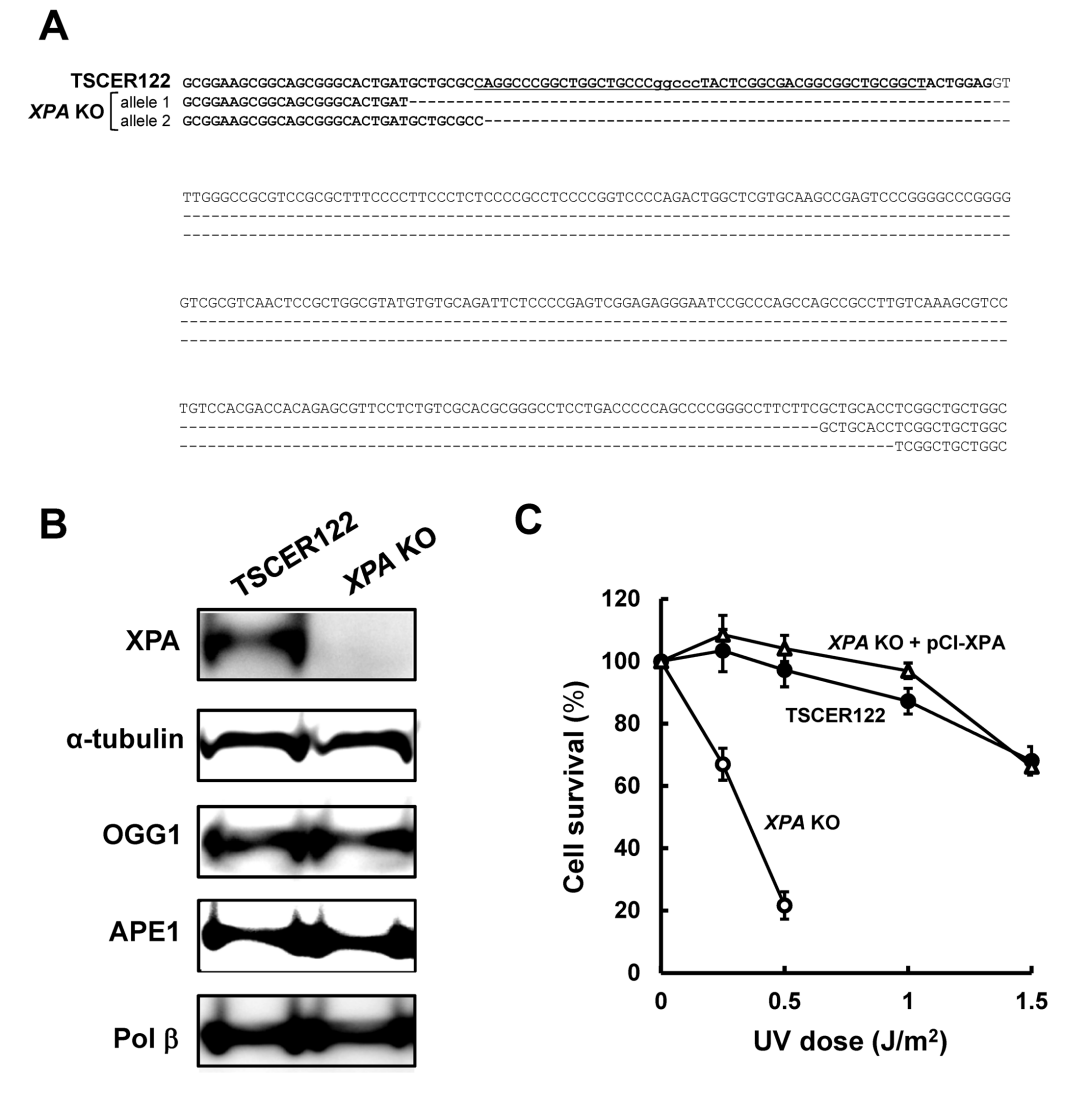
Genome sequence, protein expression, and phenotype of *XPA* knockout cells. (A) Genome sequence around the *XPA* locus in TSCER122 and *XPA* KO cells. The sequence in bold indicates a part of exon 1 of the *XPA* gene. The underlined sequence indicates the target site of ZFN. “−” indicates a one-base deletion. *XPA* KO cells carry 308-bp deletions at the *XPA* locus in both the alleles. (B) Western blot analysis for XPA, OGG1, APE1, and Pol β protein. Whole cell extracts from TSCER122 and *XPA* KO cells were loaded onto a 10% SDS-polyacrylamide gel. α-Tubulin served as the internal control. (C) Survival of TSCER122 (closed circles) and *XPA* KO (open circles), and *XPA* KO + pCl-XPA (open triangles) cells after exposure to UV light. Values presented are means ± S.E.M. of three independent experiments. Experiments were performed as described in Materials and Methods.

The *XPA* gene is located on chromosome 9 and contains six exons. To knock out *XPA* in human lymphoblastoid TSCER122 cells, a customized ZFN was designed to target exon 1 of *XPA*. The target sequence of ZFN is shown in Fig 2A. After transfecting ZFN mRNA into TSCER122, we obtained one homozygous disrupted clone. The resulting *XPA* KO cells had a 308-bp deletion in exon 1 of both the alleles (Fig 2A). The protein expression was evaluated by Western blot analysis with anti-XPA monoclonal antibody (Fig 2B), showing that XPA protein was expressed in TSCER122 but not in *XPA* KO cells. OGG1, APE1, and Pol β were also detected in both TSCER122 and *XPA* KO cells, which indicates that the disruption of *XPA* did not significantly alter the expression level of BER components. *XPA* protects cells against UV-induced cytotoxicity [34]; accordingly, we compared the sensitivity of TSCER122 and *XPA* KO cells to UV irradiation. As shown in Fig 2C, *XPA* KO cells were hypersensitive to UV irradiation. Such hypersensitivity against UV was rescued by expressing the wild-type *XPA* in *XPA* KO (*XPA* KO + pCl-XPA) cells. The population doubling time of *XPA* KO cells (13 ± 0.083 h) was similar to that of TSCER122 cells (13 ± 0.047 h), indicating that *XPA* knockout did not markedly influence the proliferation rate of TSCER122 cells.

### Mutagenic events induced by oxidative DNA adducts in TSCER122 cells

We prepared targeting vectors pvIT^G^, pvIT^1x8oG^, pvIT^2x8oG^, and pvINT^2x8oG^, which carried no DNA adduct, a single 8-oxoG in the TS, tandem 8-oxoG in the TS, and tandem 8-oxoG in the NTS, respectively (refer to Materials and Methods and S1A–S1C Fig) as previously reported [30]. An overview of the TATAM system is shown in Fig 1. First, the mutant proportion induced by integration of each targeting vector was determined in TSCER122 cells. As shown in the eighth column of Table 1, the total proportion of mutants induced by pvIT^1x8oG^ was 8.1%, which was approximately 6-fold higher than that induced by the control vector pvIT^G^ (1.3%). The total proportion of mutants induced by pvIT^2x8oG^ (7.4%) was comparable with that induced by pvIT^1x8oG^, although the number of 8-oxoG in pvIT^2x8oG^ was twice that in pvIT^1x8oG^. In contrast, the mutant proportion induced by pvINT^2x8oG^ (11%) was somewhat higher than that induced by pvIT^2x8oG^. This was an unexpected result because the mutant proportion by a single 8-oxoG has been reported to be similar between TS and NTS [30].

**Table 1:** Mutation spectra induced by integration of pvIT^G^, pvIT^1x8oG^, pvIT^2x8oG^, and pvINT^2x8oG^.

Targeting vector				Single point mutation at 8-oxoG^*a*^								
	Cell	*TK* revertants analyzed	DNA adducts-integrated revertants	T	C	A	Del^*b*^	Ins^*c*^	Tandem mutations^*d*^	Non-targeted^*e*^	Total mutation	ND^*f*^
pvIT^G^	TSCER122	264	236 (100%)	0	0	0	0	0	0	3 (1.3%)	3 (1.3%)	1
	XPA KO	260	227 (100%)	0	0	0	0	0	0	3 (1.3%)	3 (1.3%)	0
pvIT^1x8oG^	TSCER122	461	422 (100%)	15 (3.6%)	5 (1.2%)	1 (0.24%)	3 (0.71%)	0	1 (0.24%)	9 (2.1%)	34 (8.1%)	3
	XPA KO	538	472 (100%)	12 (2.5%)	11 (2.3%)	3 (0.64%)	1 (0.21%)	0	0	9 (1.9%)	36 (7.6%)	3
pvIT^2x8oG^	TSCER122	713	649 (100%)	19 (2.9%)	11 (1.7%)	2 (0.31%)	0	1 (0.15%)	8 (1.2%)	7 (1.1%)	48 (7.4%)	4
	XPA KO	816	703 (100%)	31 (4.4%)	6 (0.85%)	5 (0.71%)	2 (0.28%)	1 (0.14%)	21 (3.0%)	20 (2.8%)	86 (12%)	3
	pCl-XPA^*g*^	541	471 (100%)	13 (2.8%)	5 (1.1%)	3 (0.64%)	0	0	7 (1.5%)	7 (1.5%)	35 (7.4%)	0
pvINT^2x8oG^	TSCER122	641	592 (100%)	35 (5.9%)	10 (1.7%)	0	2 (0.34%)	1 (0.17%)	12 (2.0%)	8 (1.4%)	68 (11%)	0
	XPA KO	633	539 (100%)	20 (3.7%)	11 (2.0%)	3 (0.56%)	0	1 (0.19%)	14 (2.6%)	14 (2.6%)	63 (12%)	0

^*a*^ A single-base substitution, one-base insertion, or one-base deletion detected at an 8-oxoG. In the cases of pvIT^**2x8oG**^ and pvINT^**2x8oG**^, single point mutation at 8-oxoG indicates a mutation detected at one 8-oxoG site.

^*b*^ One-base deletion.

^*c*^ One-base insertion.

^*d*^ Multiple base substitutions, deletions, and/or insertions detected at sites, including 8-oxoG, in 12 bp around the *Bss*SI site.

^*e*^ Mutations found at sites other than 8-oxoG loci.

^*f*^ Not detectable because of too low signal strength.

^*g*^ *XPA* KO + pCl-XPA cells.

### Effect of knockout of XPA on mutagenic events induced by a single 8-oxoG

**Fig 3:**
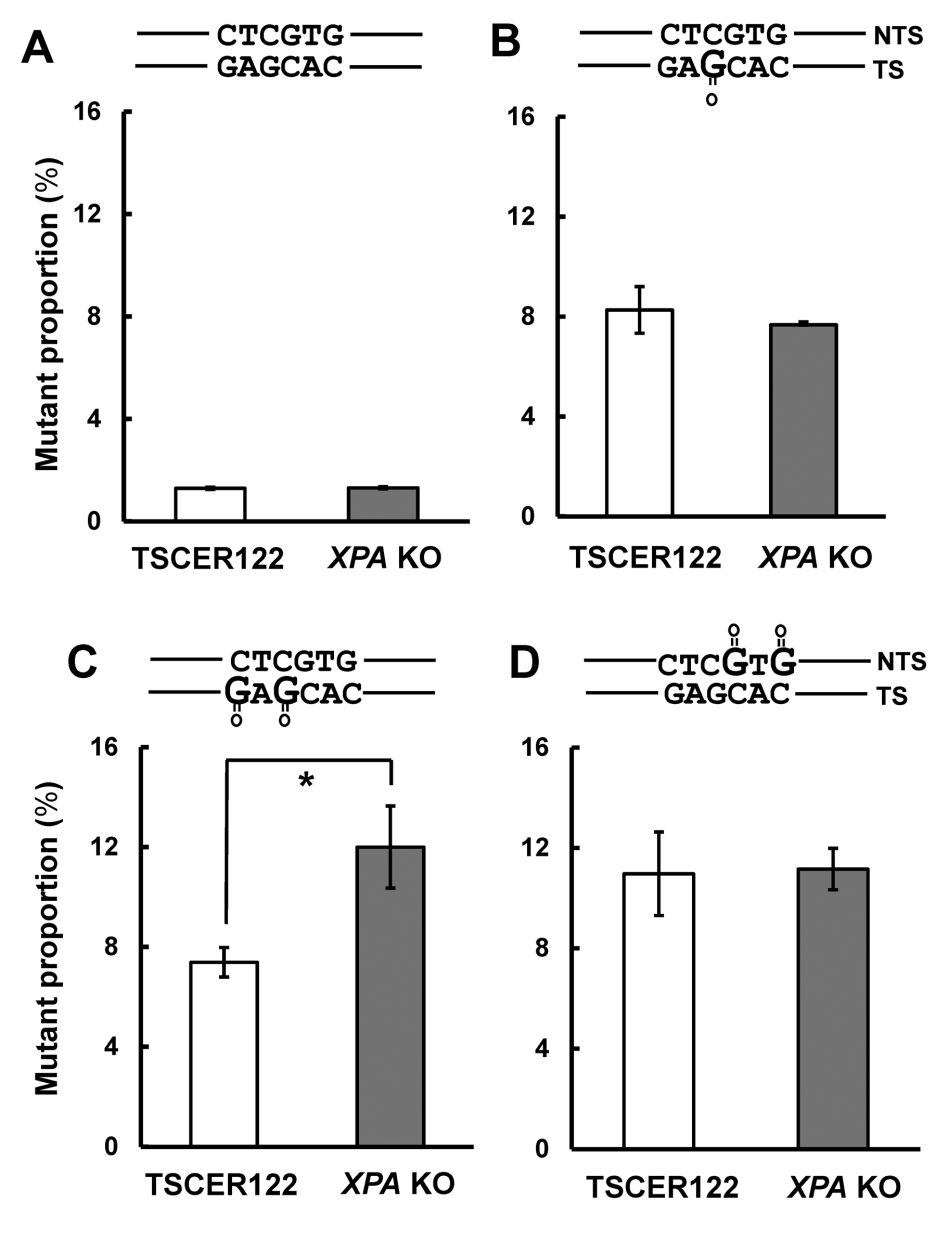
Mutant proportions induced by integration of pvIT^1x8oG^, pvIT^2x8oG^, and pvINT^2x8oG^. Mutant proportions induced by integration of (A) pvIT^G^, (B) pvIT^1x8oG^, (C) pvIT^2x8oG^, and (D) pvINT^2x8oG^ in TSCER122 and *XPA* KO cells. Values presented are the means ± S.E.M. of two independent transfections for pvIT^G^ or pvIT^1x8oG^, four independent transfections for pvIT^2x8oG^, and five independent transfections for pvINT^2x8oG^. Asterisks indicate a significant difference between TSCER122 and *XPA* KO cells (Student’s *t*-test, P < 0.05).

Next, we analyzed the effect of *XPA* knockout on the mutant proportions induced by integration of unmodified vector or a single 8-oxoG. First, the mutant proportion induced by pvIT^G^ integration was not altered by *XPA* disruption (1.3 ± 0.061 and 1.3 ± 0.043% in TSCER122 and *XPA* KO cells, respectively) (Fig 3A). Second, as shown in Fig 3B, the proportion of mutants associated with pvIT^1x8oG^ integration was also similar between TSCER122 and *XPA* KO cells (8.3 ± 0.93 and 7.6 ± 0.016%, respectively). In addition, we analyzed mutation spectra that was induced by an 8-oxoG. As shown in the fifth column of Table 1, the mutation most predominantly observed was G·C to T·A transversion at the 8-oxoG site in both TSCER122 and *XPA* KO cells. Smaller numbers of G·C to C·G transversions, G·C to A·T transitions, and one-base deletions at the 8-oxoG position and “non-targeted” mutations, which were detected at sites other than 8-oxoG, were also observed in both the cells, which were similar to results of earlier studies [30]. No significant increase in tandem mutations was observed in *XPA* KO cells (sixth column of Table 1 and Table 2). Overall, *XPA* disruption did not markedly affect mutagenesis that was induced by an isolated 8-oxoG in the genome.

**Table 2:** Spectra of single and tandem mutations induced by integration of pvIT^1x8oG^, pvIT^2x8oG^, and pvINT^2x8oG^.

		pvIT^1x8oG^		pvIT^2x8oG^			pvINT^2x8oG^	
		5′-TCCCAC8AGGCT-3′^*b*^		5′-TCCCAC8A8GCT-3′			5′-TCCCACGAGGCT-3′	
		3′-AGGGTGCTCCGA-5′		3′-AGGGTGCTCCGA-5′			3′-AGG8T8CTCCGA-5′	
Mutation^*a*^		TSCER122	XPA KO	TSCER122	XPA KO	pCl-XPA^*c*^	TSCER122	XPA KO
	5′-TCCCACGAGGCT (original)							
Single	5′-TCC**CAC** **T** **AG**GCT	15 (3.6%)	12 (2.5%)	9 (1.4%)	14 (2.0%)	4 (0.85%)	0	0
	5′-TCC**CAC** **C** **AG**GCT	5 (1.2%)	11 (2.3%)	8 (1.2%)	3 (0.43%)	3 (0.64%)	0	0
	5′-TCC**CAC** **A** **AG**GCT	1 (0.24%)	3 (0.64%)	1 (0.15%)	4 (0.57%)	3 (0.64%)	0	0
	5′-TCC**CAC** **Δ** **AG**GCT	3 (0.71%)	1 (0.21%)	0	2 (0.28%)	0	0	0
	5′-TCC**CACGA** **T**GCT	0	0	10 (1.5%)	17 (2.4%)	9 (1.9%)	0	0
	5′-TCC**CACGA** **C**GCT	0	0	3 (0.46%)	3 (0.43%)	2 (0.42%)	0	0
	5′-TCC**CACGA** **A**GCT	0	0	1 (0.15%)	1 (0.14%)	0	0	0
	5′-TCC**CAC** **C** **GAG**GCT	0	0	1 (0.15%)	0	0	0	0
	5′-TCC**CACGAG** **G**GCT	0	0	0	1 (0.14%)	0	0	0
	5′-TCC**A** **ACGAG**GCT	0	0	0	0	0	2 (0.34%)	8 (1.5%)
	5′-TCC**G** **ACGAG**GCT	0	0	0	0	0	5 (0.84%)	2 (0.37%)
	5′-TCC**T** **ACGAG**GCT	0	0	0	0	0	0	1 (0.19%)
	5′-TCC**Δ** **ACGAG**GCT	0	0	0	0	0	2 (0.34%)	0
	5′-TCC**CA** **A** **GAG**GCT	0	0	0	0	0	33 (5.6%)	12 (2.2%)
	5′-TCC**CA** **G** **GAG**GCT	0	0	0	0	0	5 (0.84%)	9 (1.7%)
	5′-TCC**CA** **T** **GAG**GCT	0	0	0	0	0	0	2 (0.37%)
	5′-TCC**CAC** **C** **GAG**GCT	0	0	0	0	0	0	1 (0.19%)
	5′-TCC**CAC** **G** **GAG**GCT	0	0	0	0	0	1 (0.17%)	0
	**Total of single**	**24 (5.7%)**	**27 (5.7%)**	**33 (5.1%)**	**45 (6.4%)**	**21 (4.5%)**	**48 (8.1%)**	**35 (6.5%)**
Tandem	5′-TCC**CAC** **T** **A** **T**GCT	0	0	3 (0.46%)	8 (1.1%)	2 (0.42%)	0	0
	5′-TCC**CAC** **C** **A** **T**GCT	0	0	1 (0.15%)	3 (0.43%)	2 (0.42%)	0	0
	5′-TCC**CAC** **T** **A** **C**GCT	0	0	1 (0.15%)	0	0	0	0
	5′-TCC**CAC** **C** **A** **C**GCT	0	0	0	0	0	0	0
	5′-TCC**CAC** **T** **A** **A**GCT	0	0	1 (0.15%)	0	0	0	0
	5′-TCC**CAC** **A** **A** **T**GCT	0	0	0	1 (0.14%)	0	0	0
	5′-TCC**CACG** **TT**GCT	0	0	0	1 (0.14%)	0	0	0
	5′-TCC**CACG** **GT**GCT	0	0	0	1 (0.14%)	0	0	0
	5′-TCC**CAC** **TTT**GCT	0	0	0	1 (0.14%)	0	0	0
	5′-TTC**CAC** **T** **A** **T**GCT	0	0	0	1 (0.14%)	0	0	0
	5′-TCC**CAC** **CC** **AG**GGT	0	0	0	0	1 (0.21%)	0	0
	5′-TCC**CACGA** **A**ACT	0	0	0	0	1 (0.21%)	0	0
	5′-TCC**CACGA** **T**ACT	0	0	0	1 (0.14%)	0	0	0
	5′-TCC**CA** **T** **GA** **C**GCT	0	0	0	1 (0.14%)	0	0	0
	5′-TCC**Δ** **AC** **T** **AG**GCT	1 (0.24%)	0	0	0	0	0	0
	5′-TCC**Δ** **ACGA** **T**GCT	0	0	0	1 (0.14%)	0	0	0
	5′-TCC**Δ** **ACGA** **C**GCT	0	0	1 (0.15%)	0	0	0	0
	5′-TCC**CA** **AΔΔ** **G**GCT	0	0	1 (0.15%)	1 (0.14%)	0	0	0
	5′-TCC**CA** **TΔΔΔ**GCT	0	0	0	1 (0.14%)	1 (0.21%)	0	0
	5′-TCC**A** **A** **A** **GAG**GCT	0	0	0	0	0	8 (1.4%)	6 (1.1%)
	5′-TCC**A** **A** **G** **GAG**GCT	0	0	0	0	0	1 (0.17%)	4 (0.74%)
	5′-TCC**G** **A** **A** **GAG**GCT	0	0	0	0	0	2 (0.34%)	2 (0.37%)
	5′-TCC**Δ** **A** **A** **GAG**GCT	0	0	0	0	0	0	1 (0.19%)
	5′-TCC**GCA** **GAG**GCT	0	0	0	0	0	1 (0.17%)	0
	5′-TCC**CA** **G** **GA** **A**GGT	0	0	0	0	0	0	1 (0.19%)
	**Total of tandem**	**1 (0.24%)**	**0 (0%)**	**8 (1.2%)**	**21 (3.0%)**	**7 (1.5%)**	**12 (2.0%)**	**14 (2.6%)**

^*a*^ Underlined sequences are observed mutations. “Δ” indicates one-base deletion.

^*b*^ “8” in the sequence indicates 8-oxoG.

^*c*^ *XPA* KO + pCl-XPA cells.

### Effect of the knockout of XPA on mutagenic events induced by tandem 8-oxoG in the transcribed or non-transcribed strand

Furthermore, we investigated the effect of *XPA* knockout on mutations caused by tandem 8-oxoG integrated in either TS or NTS. The mutant proportion induced by pvIT^2x8oG^ integration was significantly higher in *XPA* KO cells (12 ± 1.6%) than in TSCER122 cells (7.4 ± 0.59%) (Fig 3C). In contrast, the proportion of mutants that were induced by pvINT^2x8oG^ integration was not increased in *XPA* KO cells (11 ± 0.83%) compared with TSCER122 cells (11 ± 1.7%) (Fig 3D). Thus, *XPA* deficiency enhanced clustered 8-oxoG-induced mutagenesis in TS but not in NTS.

We then analyzed the mutation spectra that was induced by tandem 8-oxoG integrated in either TS or NTS. As shown in the fifth column of Table 1, the most frequent mutation was a single G·C to T·A transversion at one of the 8-oxoG positions in either TS or NTS, in both TSCER122 and *XPA* KO cells. Lesser proportions of single G·C to C·G transversions, G·C to A·T transitions, or one-base insertions/deletions were also detected irrespective of *XPA* expression. Interestingly, the proportion of tandem mutations was higher in *XPA* KO cells (3.0%) than in TSCER122 cells (1.2%) in TS (Fisher’s exact test, P < 0.05), although that proportion was comparable between TSCER122 and XPA KO cells in NTS (2.0% and 2.6%, respectively). As shown in Table 2, the tandem mutations most predominantly observed were GNG to TNT in both TSCER122 (0.46 and 1.4% in TS and NTS, respectively) and *XPA* KO cells (1.1 and 1.1% in TS and NTS, respectively). Smaller numbers of different types of tandem mutations were also detected. Unexpectedly, the proportion of non-targeted mutations was significantly higher in *XPA* KO cells (2.8%) than in TSCER122 cells (1.1%) in TS (Fisher’s exact test, P < 0.05) (seventh column of Table 1 and S1 Table). Notably, the proportion of non-targeted mutations was not significantly altered between TSCER122 and *XPA* KO cells when pvIT^G^, pvIT^1x8oG^, or pvINT^2x8oG^ was integrated into the genome.

To verify that *XPA* was actually involved in the suppression of tandem 8-oxoG-induced mutations in TS, the mutant proportion induced by pvIT^2x8oG^ integration was examined in *XPA* KO + pCl-XPA cells. As shown in the eighth column of Table 1, the total mutant proportion was comparable between TSCER122 and *XPA* KO + pCl-XPA cells (7.4% and 7.4%, respectively). Similarly, the proportions of tandem mutations and non-targeted mutations in *XPA* KO + pCl-XPA cells (1.5% and 1.5%, respectively) were as low as those detected in TSCER122 cells (1.2% and 1.1%, respectively) (Table 2 and S1 Table).

The targeting efficiencies of the vectors to *XPA* KO cells were similar to those to TSCER122 cells (data not shown), suggesting that *XPA* disruption did not influence homologous recombination efficiency.

## Discussion

Because clustered DNA adducts are poorly repairable by BER [8–11, 35], we postulated that an alternative repair machinery is involved in the removal of DNA adducts *in vivo*. To investigate the role of NER on the oxidative DNA damage-induced mutagenesis, *XPA* can be a good candidate gene to be examined without altering the expression of BER proteins (Fig 2B), whereas some other NER components change the status of OGG1 [36, 37]. Loss of *XPA* does not sensitize cells to IR [38]. *XPA* disruption in TSCER122 cells does not alter cell survival after IR irradiation (data not shown). However, decreased *XPA* expression results in increased IR-induced chromosomal aberrations levels [39], implying the importance of *XPA*-mediated NER in repairing IR-induced DNA damage. However, the biological relevance of NER and IR-induced mutagenesis has remained unclear. In this study, we present evidence that NER plays a substantial role in the suppression of mutations that were induced by clustered oxidative DNA adducts in human cells.

Our results indicate that NER is not crucial for suppressing mutations caused by a single 8-oxoG (Fig 3B). This observation may be because of the abundant repair activities of BER proteins, such as OGG1, MYH [30], and Nei endonuclease VIII-like glycosylases [40], to remove 8-oxoG in cells. Alternatively, the small helix distortion due to a single 8-oxoG may not be enough for efficient recognition by NER [41].

The observation that NER disruption significantly increased the proportion of mutants that were induced by tandem 8-oxoG in TS but not in NTS (Fig 3C and 3D) is consistent with the results of a previous study that revealed transcription coupled repair of 8-oxoG [28]. In fact, in TSCER122 cells, the mutant proportion of tandem 8-oxoG in NTS was higher than that in TS (sixth column of Table 1). This enhanced mutagenesis of tandem 8-oxoG is probably due to delayed BER at the tandem DNA adducts [11]. In addition, at the site of clustered DNA adducts, the binding of DNA glycosylase on one DNA adduct inhibits repair of the neighboring damage [35].

NER is most likely the most efficient mechanism to repair clustered DNA adducts because of its capability to remove all adducts at once [42]. Furthermore, it is plausible that NER removes clustered 8-oxoG by processing the BER intermediate [43]; once DNA glycosylase cleaves one of the 8-oxoG at the tandem site, a resulting AP site can initiate NER-mediated repair of clustered DNA damage. Obviously, NER proteins recognize 8-oxoG and also facilitate tandem DNA adducts containing an AP-site analog in vitro [27, 42], indicating that both 8-oxoG and its BER intermediate in the clustered DNA damage attract NER. Moreover, the unique structural changes within the DNA helix induced by complex damage may contribute to the damage recognition by NER [44].

As shown in the sixth and seventh column of Table 1, the enhanced mutagenesis of tandem 8-oxoG in TS in *XPA* KO cells is because of the increased proportions not only of tandem mutations but also of non-targeted mutations, which are induced near the adduct position. Although the exact reason for the increased proportion of non-targeted mutations in *XPA* KO cells remains unclear, it may be because of stalled replication or retarded repair of sites of clustered DNA adducts in *XPA* absence.

It is noteworthy that IR produces a variety of DNA adducts, which are formed in close proximity in one or both DNA strands. NER may be involved in repairing different types of clustered DNA damage, which can lead to slow or stalled BER, thus protecting the genome from the lethal strand break formation. Further studies that examine how NER contributes to repair of different kinds of clustered DNA damage will help in understanding its functions in IR-induced DNA damage.

We conclude that NER suppresses mutations induced by clustered oxidative DNA adducts. To the best of our knowledge, this is the first report providing the *in vivo* evidence that NER is involved in the repair of clustered oxidative DNA damages. This role for NER may contribute to the oxidative stress leading to neurodegeneration in patients with *XPA* [45].

## Supporting Information

S1 Fig Details of the site of 8-oxoG. The 8-oxoG site for (A) pvIT^1x8oG^, (B) pvIT^2x8oG^, and (C) pvINT^2x8oG^. The position of an 8-oxoG is indicated by “8” in the primer sequence. A single 8-oxoG or tandem 8-oxoG were inserted at the *Bss*SI site. The MseI^R^ site was placed near the site in the 3F and 2R primers.
(PPT)

S1 Table The spectra of non-targeted mutations induced by integration of pvIT^G^, pvIT^1x8oG^, pvIT^2x8oG^, and pvINT^2x8oG^. ^*a*^ Underlined sequences are observed mutations. “Δ” indicates one-base deletion. del., deletion; ins., insertion. ^*b*^ “8” in the sequence indicates 8-oxoG. ^*c*^ *XPA* KO + pCl-XPA cells.
(XLSX)
