# Supplementary material for: Xeroderma Pigmentosum Group A Suppresses Mutagenesis Caused by Clustered Oxidative DNA Adducts in the Human Genome
Source: PLoS One. 2015 Nov 11;10(11):e0142218. doi: 10.1371/journal.pone.0142218 (PMC4641734; doi:10.1371/journal.pone.0142218)
Supplement: S1 Fig — The 8-oxoG site for (A) pvIT1x8oG, (B) pvIT2x8oG, and (C) pvINT2x8oG. The position of an 8-oxoG is indicated by “8” in the primer sequence. A single 8-oxoG or tandem 8-oxoG were inserted at the BssSI site. The MseIR site was placed near the site in the 3F and 2R primers. (PPT) [file pone.0142218.s001.ppt]

## Slide 1
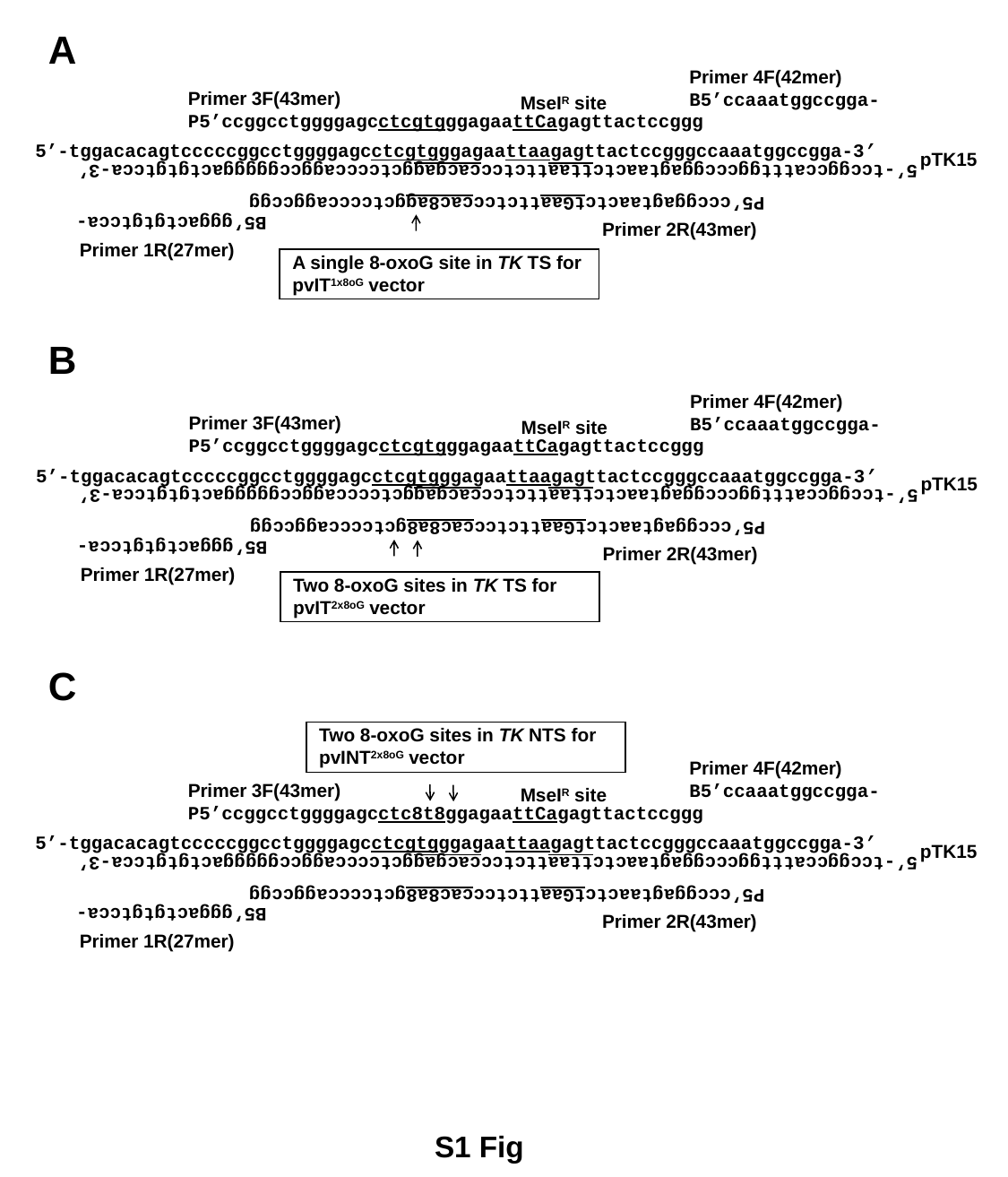

A
Primer 4F(42mer)
B5’ccaaatggccgga-
Primer 3F(43mer)
P5’ccggcctggggagcctcgtgggagaattCagagttactccggg
MseIR site
5’-tggacacagtcccccggcctggggagcctcgtgggagaattaagagttactccgggccaaatggccgga-3’
pTK15
5’-tccggccatttggcccggagtaactcttaattctcccacgaggctccccaggccgggggactgtgtcca-3’
P5’cccggagtaactctGaattctcccac8aggctccccaggccgg
B5’gggactgtgtcca-
Primer 2R(43mer)
Primer 1R(27mer)
A single 8-oxoG site in TK TS for pvIT1x8oG vector
B
Primer 4F(42mer)
B5’ccaaatggccgga-
Primer 3F(43mer)
P5’ccggcctggggagcctcgtgggagaattCagagttactccggg
MseIR site
5’-tggacacagtcccccggcctggggagcctcgtgggagaattaagagttactccgggccaaatggccgga-3’
pTK15
5’-tccggccatttggcccggagtaactcttaattctcccacgaggctccccaggccgggggactgtgtcca-3’
P5’cccggagtaactctGaattctcccac8a8gctccccaggccgg
B5’gggactgtgtcca-
Primer 2R(43mer)
Primer 1R(27mer)
Two 8-oxoG sites in TK TS for pvIT2x8oG vector
C
Two 8-oxoG sites in TK NTS for pvINT2x8oG vector
Primer 4F(42mer)
B5’ccaaatggccgga-
Primer 3F(43mer)
P5’ccggcctggggagcctc8t8ggagaattCagagttactccggg
MseIR site
5’-tggacacagtcccccggcctggggagcctcgtgggagaattaagagttactccgggccaaatggccgga-3’
pTK15
5’-tccggccatttggcccggagtaactcttaattctcccacgaggctccccaggccgggggactgtgtcca-3’
P5’cccggagtaactctGaattctcccac8a8gctccccaggccgg
B5’gggactgtgtcca-
Primer 2R(43mer)
Primer 1R(27mer)
S1 Fig
